# Identification and Quantification by Targeted Metabolomics of Antibiotic-Responsive Urinary Small Phenolic Molecules Derived from the Intestinal Microbiota in Mice

**DOI:** 10.20411/pai.v4i1.284

**Published:** 2019-03-14

**Authors:** Mark E. Obrenovich, George E. Jaskiw, Renliang Zhang, Belinda Willard, Curtis J. Donskey

**Affiliations:** 1 Pathology and Laboratory Medicine Service; Louis Stokes Cleveland Department of Veterans Affairs Medical Center; Cleveland, Ohio; 2 Research Service; Louis Stokes Cleveland Department of Veterans Affairs Medical Center; Cleveland, Ohio; 3 Department of Chemistry; Case Western Reserve University; Cleveland, Ohio; 4 Department of Medicinal and Biological Chemistry; College of Pharmacy and Pharmaceutical Sciences; University of Toledo; Toledo, Ohio; 5 Psychiatry Service, Louis Stokes Cleveland Department of Veteran's Affairs Medical Center, Cleveland, Ohio; 6 School of Medicine; Case Western Reserve University; Cleveland, Ohio; 7 Proteomics and Metabolomics Core; Lerner Research Institute; Cleveland Clinic Foundation; Cleveland, Ohio; 8 Geriatric Research, Education and Clinical Center, Louis Stokes Cleveland Department of Veterans Affairs Medical Center, Cleveland, Ohio

**Keywords:** phenolic, microbiota, schizophrenia, autism, anaerobes, mice, clindamycin, aztreonam, piperacillin/tazobactam, antibiotics

## Abstract

**Background:**

Urinary levels of small molecules generated by the gut microbiota (GMB) constitute potential biomarkers for the state of the GMB. Such metabolites include numerous small phenolic molecules linked to anaerobic bacteria, particularly *Clostridium* species. Due to multiple technical challenges, however, the relationship between these chemicals and the GMB remains poorly characterized. Improved, high-performance liquid chromatography-mass spectrometry (LC-MS)-based metabolomic analysis can now reliably separate and quantify low levels of multiple small phenolic molecules and their structural isomers.

**Methods:**

CF-1 (female mice) were treated over 2 consecutive days with either i) vehicle, ii) one of 2 different antibiotic regimens (clindamycin or piperacillin/tazobactam) known to inhibit intestinal anaerobes and promote colonization by *Clostridium difficile* and other pathogens or iii) an antibiotic (aztreonam) that suppresses facultative Gram-negative bacteria but not *enterococci* or anaerobes and does not promote pathogen colonization Urine collected 24 hours after the last treatment was analyzed by LC-MS.

**Results:**

We identified over 25 compounds, many of which had not been previously reported in mouse urine. Eleven small phenolic molecules showed significant antibiotic-related changes. Urinary levels of the hydroxyphenylpropionic acids were suppressed by clindamycin and pipera-cillin/tazobactam treatment, but were elevated in aztreonam-treated mice. In addition, aztreonam treatment was associated with elevated levels of the dihydroxyhydrocinnamic acids.

**Conclusions:**

Profiles of differential changes in urinary small phenolic molecules may provide an index of anaerobic bacterial species in the GMB and could prove useful in monitoring susceptibility to overgrowth of pathogens such as *C. difficile*.

## INTRODUCTION

Numerous small molecules (< 2,000 Da) produced by the gut microbiota (GMB) [1, 2] enter the systemic circulation and are excreted in urine [3]. Identifying these chemicals and clarifying their underlying kinetics may facilitate development of biomarkers relevant to GMB-sensitive diseases. For example, alteration of the GMB by antibiotics may facilitate colonization by and overgrowth of healthcare-associated pathogens such as *Clostridium difficile* and vancomycin-resistant enterococci (VRE) [4–6]. *C. difficile* is the most common pathogen identified as a cause of healthcare-associated infections in the United States, resulting in substantial morbidity, mortality, and economic burden [7]. Having an index for vulnerability or conversely resistance to colonization by pathogens could be clinically useful. To that end, we recently demonstrated that several GMB-as-sociated urinary chemicals were affected by antibiotic treatment in a mouse model of colonization resistance (ie, a host defense provided by the indigenous microbiota to inhibit colonization and overgrowth of potentially pathogenic microorganisms) [8].

The urinary metabolome includes numerous small phenolic molecules (SPMs) that can either be synthesized from L-phenylalanine (L-PHE) or L-tyrosine (L-TYR) [9, 10] or derived from the biotransformation of polyphenols or related dietary constituents [11]. Anaerobic bacteria such as *Clostridium* species, including *C. difficile,* have been implicated in all these routes [11–15]. Furthermore, many of the same SPMs have been identified as potential biomarkers in neuropsychiatric conditions such as schizophrenia and autism [12–15]. Accordingly, our first step was to determine which of the SPMs are affected by targeted perturbations of the GMB.

Liquid chromatography (LC) coupled with mass spectrometry (MS) offers high sensitivity and resolution yet relatively simple sample preparation, and it is now the preferred platform for both targeted and non-targeted metabolomic studies [16]. Even this approach, however, has shortcomings. A prototypical SPM of interest, 3-hydroxy-3-(3-hydroxyphenyl) propanoic acid (3,3-HPH-PA), has 12 structural isomers (C_9_H_10_O_4_) differing in the position of a single hydroxyl group [17]. The isomers exhibit similar retention times and mass spectra and hence can easily be misidentified by LC/MS [13, 18]. In addition, many SPMs are present in mammalian compartments as mixtures of free chemical and various conjugates [11, 19]. In response, we recently developed an LC-MS protocol that addresses these challenges and supports high resolution and accurate quantification of multiple SPMs [17]. We have now applied this assay to the SPM urinary metabolome of mice with a diverse intestinal microbiota used in a mouse model of colonization resistance [4]. Our primary hypothesis was that urinary SPMs would be differentially affected by antibiotics that do versus do not suppress intestinal anaerobes and promote colonization by healthcare-associated pathogens.

## MATERIALS AND METHODS

### Animals

The study protocol was approved by the Cleveland VA Medical Center's Institutional Animal Care and Use Committee. Female CF-1 mice weighing 25 to 30 g (Harlan Sprague-Dawley, Indianapolis, IN) were housed in groups (n = 5) and fed a sterilized Teklad Global 18% protein-extruded rodent diet (Harlan Teklad, Madison, WI). Groups received daily subcutaneous injections (0.1 mL total volume) of saline, clindamycin (14 mg/day), piperacillin/tazobactam (8 mg/day), or aztreonam (3 mg/day) for 2 consecutive days. The doses were equivalent to clinical doses administered to patients over a 24-hour period (mg/g body weight).

Clindamycin and piperacillin/tazobactam were studied because they have previously been shown to significantly suppress anaerobes in stool and promote colonization by and overgrowth of healthcare-associated pathogens, including *C. difficile,* VRE, and *Klebsiella pneumoniae* [5, 6] (Supplementary Table 1). Clindamycin inhibits anaerobes but not indigenous enterococci or facultative gram-negative bacilli (eg, *Escherichia coli*), whereas piperacillin/tazobactam has broad-spectrum activity resulting in inhibition of indigenous enterococci and facultative gram-negative bacilli in addition to anaerobes [5]. Aztreonam was studied as a comparator because it does not suppress anaerobes in stool or promote pathogen colonization [4, 5]. Aztreonam inhibits indigenous facultative gram-negative bacilli but not enterococci [5].

On day 3, when antibiotic-induced changes in GMB composition were expected to be maximal [6, 8], individual mice were placed in clean cages with no bedding and observed continuously for up to 6 periods of 1 hour. Fresh urine specimens were collected by pipette tip and transferred to sterile Eppendorf tubes. Our preliminary experiments demonstrated that even transient contact of urine with stool could change metabolite concentration in the urine. For that reason, urine specimens in contact with stool pellets were not collected and mice were moved into new clean cages as needed to avoid contamination of urine by stool [8]. Urine specimens were centrifuged at 14,000*g* for 15 minutes at 8°C, digested with beta-glucuronidase and aryl sulfatase for 24 hours according to the manufacturer's recommendations (Roche Diagnostics GmbH, Mannheim, Germany) as previously described [17], and then frozen at −80°C until being thawed for analysis [8, 17].

### Chemicals and Reagents

Antibiotics were USP grade (Pfizer, New York). All chemicals and reagents were of the highest purity and grade commercially available. Chemical standards for 3,3-HPHPA and internal standard (2-(Benzoylamino) acetic Acid-d5) (HA-d5) were purchased from Toronto Research Chemicals (North York, Canada), and 3-(2,3-dihydroxyphenyl)propanoic acid (2,3-DHHCA) was procured from Santa Cruz Biotechnology (Santa Cruz, CA). All other standards were purchased from Sig-ma-Aldrich Co. LLC. (St. Louis, MO). LC-MS grade solvents; water, formic acid, and acetonitrile (ACN) were purchased from Fisher Scientific (Pittsburgh, PA).

A standard stock solution containing all compounds of interest and the internal standards was prepared by accurate weighing of each compound (1 mg/mL) in suitable solvent and initially blanketed with nitrogen in glass vials equipped with Teflon^®^-lined screw caps and stored at −20°C. The stock solution was serially diluted to obtain working solutions in the range 10 ng/mL to 2000 ng/mL. To generate standard curves over a range of concentrations for quantitation, we used charcoal-stripped urine for the matrix (IRISpec CA/CB reagent, Beckman Coulter, Chatsworth, CA). Both reagents were suitable, and in this case CB reagent was used as the matrix, when exploring matrix effects in urine. Protein was not precipitated, but all cells were eliminated with a clinical spin and prepared the same way as urine samples. In brief, 10 μL of the internal standard at 1000 ng/mL and 50 μL of matrix were added to each diluted standard solution at the final volume of 100 μL (for details see [8]).

### Urine Sample Preparation for LCMS

Urine samples were stored at −80°C until thawed for analysis. After aliquoting 100 μL of urine, internal standards in water were added, and any protein in samples was precipitated with 6-fold volume of ice-cold acetonitrile by vortexing for 1 minute, and followed by recovery of the supernatant following centrifugation at 14,000 g at 5°C for 15 minutes. After drying (Speed vacuum), samples were reconstituted to 100 μL of which 20 μL was injected for LC–MS analysis. When digestion was required, supernatants were transferred to a new 1.5-mL Eppendorf tube and digested with aryl sulfatase and beta glucuronidase (from Helix pomatia, Roche Applied Science, Mannheim Germany) per manufacturer instructions [17, 20]. After digestion, the samples were centrifuged (14,000 g × 10 minutes). The supernatant was transferred to a clean HPLC injection vial for LCMS analysis. Sample metabolites were previously evaluated for interfering chemical species known to exert quenching or matrix effects [21]. We previously demonstrated that once the issue of potential conjugates was addressed, urine samples required minimal additional pretreatments (eg, with sulfatase, glucuronidase) [8]. Preliminary stability studies were evaluated using pooled samples in triplicate. Freeze-thaw stability was assessed for 4 cycles from −80°C to room temperature. Benchtop stability was tested by analyzing samples, which were left out on the bench top at room temperature for more than 8 hours before analysis.

### Compound Identification and Metabolite Analysis

The initial analysis was conducted at the Cleveland Clinic Small Molecule Mass Spectrometry Core on a Shimadzu LCMS-8050 (Kyoto Japan). Compound ions were targeted for collision-induced dissociation (CID) fragmentation based on the previously determined accurate mass and retention time of standards [8, 17]. Analytes were identified by their LC retention time and by monitoring their specific multiple reaction monitoring (MRM) transitions. Selectivity was evaluated by injecting a single analyte, while measuring MRM transitions of all analytes under consideration. These transitions are described in our previous report [17]. Typically, 1 quantitation MRM transition is collected along with several confirmatory ion pairs using a minimum of 2 transitions for each compound, where possible.

A reconstituted sample of 20 μL was injected onto a Shimadzu 8050LCMS system (Shimadzu Kyoto, Japan) with a Luna, C18 (3 μm, ODS 100A, 2 × 150 mm) LC column from (Phenomenex, Rancho Palos Verdes, CA). Eluents A (water + 0.2% acetic acid) and B (acetonitrile + 0.2% acetic acid) were used under gradient conditions at a flow rate of 0.3 mL/min. The gradient program started with isocratic elution with 100% A (0-2 minutes, followed by 100% A to 100% B; 2-8 minutes; 8-18 minutes, 100% B, 18-18.5 minutes, 100% B to 100% A; 18.5-26 minutes, 100% A). This permitted the separation of over 50 analytes. Then 5 μL of HPLC column effluent was introduced onto a Shimadzu 8050 triple quadruple MS system (Shimadzu, Kyoto, Japan) and analyzed using either positive or negative electrospray ionization (ESI) in the MRM mode [17] with the following parameters: nebulizing gas flow 3 L/min, heating gas flow 10 L/minutes, drying gas flow 10 L/minutes, interface temperature 300°C, DL temperature 250°C, heat block temperature 400°C, and argon as the collision-induced dissociation (CID) gas. Retention time was determined during method development and the fragmentation patterns for urine metabolite unknowns and authentic standard compounds were compared after an initial optimization of the collision energy [17]. Internal standard calibration curves were used to quantify metabolites. The use of isotope dilution LC-MS for metabolic profiling is the standard method for quantification of rodent and bacterial metabolites or cellular material [22].

Quantitation was performed using the internal standard calibration method. Each calibration sample was assayed in duplicate over 3 different days. The peak area of each analyte was divided by the peak area of the corresponding internal standard. This ratio was then plotted on the y-axis against known quantities of each analyte to generate calibration curves by linear regression. Whenever possible we used isotope dilution mass spectrometry and standards with a minimum of 3 mass units difference relative to the native compound. Where no isotope was commercially available, we used unlabeled compounds that were structurally similar to the compound of interest. The sensitivity of this method was based on the limits of detection and limits of quantification values for each analyte, which were determined at a signal-to-noise ratio of 3 and 10, respectively. To evaluate the carry-over effect, a blank of methanol and water was injected after the highest concentration standard and after each set of standards and throughout the runs every 20 injections.

### Urinary Creatinine Normalization

Urinary metabolites were normalized using creatinine and the d3-creatinine internal standard as previously described [17]. This required special handling and hydrophilic interaction liquid chromatography (HILIC). HILIC employs a multimodal partitioning technique, where highly polar analytes are retained on the column by partitioning between a water-enriched layer attracted by the polar stationary phase and the solvent, consisting of a mixture of acetonitrile and aqueous buffer in the range of 10% to 90%. Creatinine was quantified using the following parameters: HIL-IC SeQuant^®^ ZIC^®^-cHILIC Column, 250 × 4.6 mm ID 3 μm particle size. Eluent C (acetonitrile) and eluent D (water with 0.1M ammonium acetate) were used across the HILIC gradient (0-2 minutes, 90% C / 10% D; 2-20 minutes, 90% C / 10% D to 10% C / 90% D; 20-21 minutes: 10% C / 90% D to 90% C / 10%; D 21-30 minutes, 90% C / 10% D) at a flow rate of 1 mL/minute. The pressure maximum was 120 to 200 bar [17].

## DATA ANALYSIS

The data were processed using LabSolutions© (Shimadzu) software. Each peak was manually checked for retention time and proper integration, after applying the auto-quantitation method. Analytes were normalized by using urine creatinine. Levels for each of the 3 antibiotic-treated groups were compared to those for control (saline-treated) using the Mann-Whitney U test. Significance was set at an alpha value of 0.05. No adjustments were made for multiple comparisons.

## RESULTS

Urinary creatinine concentrations (569 ± 135 nmol/mL) in controls did not differ significantly from that of any other antibiotic-treated group. Accordingly, the concentration of each metabolite was normalized by the individual creatinine value. Table 1 shows the concentrations of the metabolites in the urine of control mice collected 24 hours after completion of 2 consecutive days of subcutaneous injections of saline. Each of the compounds was detected in the urine of control mice. The compounds included 11 that to our knowledge had not been previously identified in mouse urine and 5 compounds that had been previously identified but not quantified in mouse urine.

**Table 1.** 

Urinary Levels in Controls on Day 3		
μmol/mmol creatinine	MEAN	STDEV
benzoic acid	0.010	0.006
3-hydroxybenzoic acid†	14.94	1.74
4-hydroxybenzoic acid	45.95	5.21
hippuric acid	433.11	139.20
3-hydroxyhippuric acid†	1.31	0.33
4-hydroxyhippuric acid	0.51	0.07
2-hydroxyphenylacetic acid	12.11	2.54
3-hydroxyphenylacetic acid†	22.29	8.98
4-hydroxyphenylacetic acid	11.35	2.48
4-hydroxyphenyllactic acid‡	62.82	20.52
4-hydroxyphenylpyruvic acid	6.44	2.02
3-phenylpropionic acid†	0.22	0.05
3,2-hydroxyphenylpropionic acid†	3.37	1.69
3,3-hydroxyphenylpropionic acid‡	139.83	71.55
3,4-hydroxyphenylpropionic acid †	3.35	1.87
3,3-hydroxyphenylhydropropionic acid†	7.67	1.43
3-hydroxycinnamic acid†	14.76	4.50
4-hydroxycinnamic acid†	82.32	26.91
3,4-dihydroxycinnamic acid‡	2.08	0.87
2,3-dihydroxyhydrocinnamic acid†	13.58	2.13
2,4-dihydroxyhydrocinnamic acid†	14.84	2.35
3,4-dihydroxyhydrocinnamic acid‡	6.48	1.27
homovanillic acid	17.13	3.78
vanillylmandelic acid	0.24	0.04
ferulic acid‡	46.79	6.93
Catechin	1.94	0.67

Levels of metabolites in mouse urine collected 24 hours after completion of 2 consecutive days of subcutaneous injections (0.1 mL) of saline.

† not previously identified

‡ previously identified but not quantified in mouse urine under baseline conditions.

**Figure 1.**
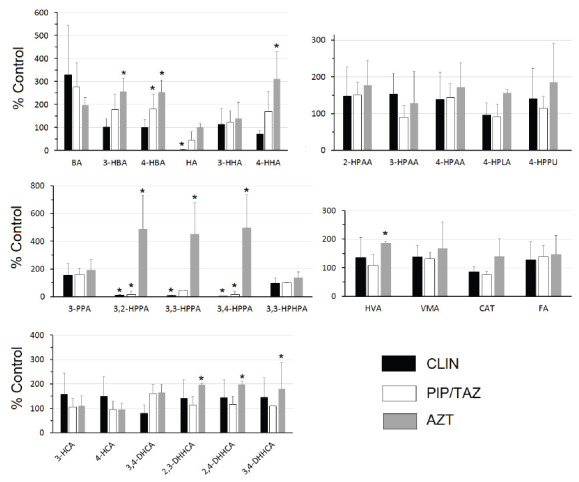
**Urinary levels expressed as % control.** Urine was collected 24 hours after completion of 2 daily SC treatments with saline (control), clindamycin (8 mg/day) (CLIN), piperacillin + tazobactam (8 mg/day) (PIP/TAZO), or aztreonam (3 mg/day) (AZT). Comparisons between each treatment and control were made by Mann-Whitney U test with statistical significance set at *P* < 0.05* (benzoic acid (BA), 3-hydroxy-benzoic acid (3-HBA), 4-hydroxybenzoic acid (4-HBA), hippuric acid (HA), 3-hydroxyhippuric acid (3-HHA), 4-hydroxyhippuric acid (4-HHA), 2-hydroxyphenylacetic acid (2-HPAA), 3-hydroxypheny-lacetic acid (3-HPAA), 4-hydroxyphenylacetic acid (4-HPAA), 4-hydroxyphenyllactic acid (4-HPLA), 4-hydroxyphenylpyruvic acid (4-HPPU), 3-phenylpropionic acid (3-PPA), 3,2-hydroxyphenylpropionic acid (3,2-HPPA), 3,3-hydroxyphenylpropionic acid (3,3-HPPA), 3,4-hydroxyphenylpropionic acid (3,4-HPPA 3,3-hydroxyphenylhydropropionic acid (3,3-HPHPA), 3-hydroxycinnamic acid (3-HCA) 4-hy-droxycinnamic acid (4-HCA), 3,4-dihydroxycinnamic acid (3,4-DHCA), 2,3-dihydroxyhydrocinnamic acid (2,3-DHHCA), 2,4-dihydroxyhydrocinnamic acid (2,4-DHHCA), 3,4-dihydroxyhydrocinnamic acid (3,4-DHHCA), homovanillic acid (HVA), vanillylmandelic acid (VMA), ferulic acid (FA), catechin (CAT).

Figure 1 shows the urine concentrations of the SPM metabolites expressed as a percentage of the concentrations in the control mice. Clindamycin treatment resulted in statistically significant reductions in HA and 3,2-HPPA, 3,3-HPPA, and 3,4-HPPA. Piperacillin/tazobactam treatment resulted in statistically significant reductions in 3,2-HPPA and 3,4-HPPA. In contrast, aztreonam treatment resulted in statistically significant increases in 3-hydroxybenzoic acid (3-HBA), 4-HBA, 4-hydroxyhippuric acid, 3,2-HPPA, 3,3-HPPA, 3,4-HPPA, HVA, and the dihydroxyhydrocinnamic acids (DHHCAs) (2,3-DHHCA, 2,4-DHHCA and 3,4-DHHCA) (Figure 1).

## DISCUSSION

Using an improved LC/MS approach we were able to quantify 25 SPMs and 1 polyphenol (cate-chin) (Table 1). Eleven of the SPMs were significantly affected by antibiotic treatment. In support of our primary hypothesis, the SPM urinary metabolites were differentially affected by antibiotics depending on their ability to suppress versus not suppress intestinal anaerobes and promote colonization by healthcare-associated pathogens. Specifically, clindamycin and piperacillin/tazobact-am are known to suppress anaerobes and treatment with these agents resulted in reductions in the group of hydroxyphenylpropionic acids. In contrast, aztreonam is known to have no or minimal impact on anaerobes, and treatment with this agent did not result in suppression of hydroxyphen-ylpropionic acids, but it promoted increases in dihydroxyhydrocinnamic acids.

There are several reasons why many of the SPMs have not previously been detected in mouse urine. First, small molecules in biological systems often exist as diverse conjugates generated by the host and/or gut microbiota. The major phase II conjugation and metabolism pathways include methylation, β-glucuronidation of hydroxyl-, carboxyl-, amino- and thiol-groups, and sulfation of hydroxyl- and amino groups [11, 19]. By conducting hydrolysis using both β-glucuro-nidase and aryl sulfatase before analyzing samples, we liberated all of a given compound irrespective of its native conjugation status, increasing the net amount of the free chemical and making it easier to measure.

Second, the family of SPMs contains many highly similar molecules. In preliminary studies, the structural isomers of 3,3-HPHPA for instance, could not be adequately resolved using a different column (Prodigy C 18 column (2 × 150 mm, Prodigy, 5μm, ODS(2), Phenomenex, Rancho Palos Verdes, CA) (data not shown). The 3,3-HPHPA peak was embedded in multiple overlapping isomeric peaks. Through a series of studies, employing new LC column technology (Luna^®^ 3 μm C18(2) 100 A LC Column 150 × 2 mm), structural and targeted metabolomic approaches across multiple platform systems, and state of the art software in conjunction with MS library searches, we eventually achieved separation and identification of important isomers and fragmentation patterns [17].

Third, the strongest confirmation of molecular identity requires availability of an authentic high purity standard. We were able to purchase commercial standards for all the chemicals we quantified. However, there were several structural isomers (3,2-HPHPA, 3,4-HPHPA, 3-hy-droxy-2-(3-hydroxyphenyl)propanoic acid), *o*-hydroxyphenyllactic acid, *m*-hydroxyphenyllactic acid, 3-hydroxyphenyllactic acid (2,3-Dihydroxy-3-phenylpropanoic acid), 3,5-DHHCA) that we could not readily obtain and hence, whose presence or absence we could not determine.

The urinary metabolome of the mouse has not been as extensively characterized as that of the rat. However, in agreement with another study in the mouse [23], HA was by far the most abundant SPM excreted (Table 1). Comparisons with the rat suggest several similarities. Levels of 3,3-HPPA exceed those of 3,4-HPPA by several-fold [24, 25] as do levels of 4-hydroxycinnamic acid relative to 3-hydroxycinnamic acid [26]; There are significant amounts of 4-hydroxyphenyllactic acid [24] (Table 1).

## GMB AND THE SMALL PHENOLIC MOLECULES

**Figure 2.**
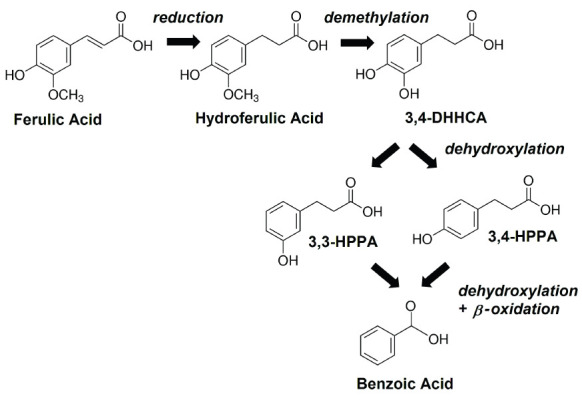
The main GMB-mediated metabolic pathway for ferulic acid in the mouse; benzoic acid (BA), 3,3-hydroxyphenylpropionic acid (3,3-HPPA), 3,4-hydroxyphenylpropionic acid (3,4-HPPA), 3,4-dihydroxyhydrocinnamic acid (3,4-DHHCA).

Oral administration of isotopically labeled L-PHE or L-TYR results in excretion of multiple labeled SPMs in man and other mammals [9, 27, 28]. The appearance of some of these, can be suppressed by pretreatment with neomycin [9], an antibiotic that is poorly absorbed from the gastrointestinal tract and hence acts only on the GMB [29]. Analogously, orally administered isotopically labeled flavonoids can be converted to numerous labeled urinary SPMs both in man and in the rodent [30–32]. Levels of many SPMs, can be eliminated or significantly lowered by prior administration of neomycin [30, 33] or other broad-spectrum antibiotic treatments [34]. These same SPMs are identified as GMB-related in studies of animals considered to be germ-free [24, 35–38] and in patients with ileostomies [39–41]. Thus, it is now well established that kinetics of some SPMs are at least in part dependent on the GMB [11, 42] (Figure 2), whereas those of other SPMs are largely mediated by endogenous processes within host cells [43].

Diet is another dominant factor that determines the GMB-dependent metabolome [11, 44]. Our mice were fed a largely plant-based laboratory diet consisting of soybean meal, brewer's dry yeast, and wheat [45]. Soybeans contain only low quantities of catechin and cinnamic acids [46] and brewer's yeast does not contain polyphenolic precursors of SPMs. Wheat and most other grains do however contain large amounts of ferulic acid (FA) [47, 48]. Less than 10% of the total FA is in the free form that can readily be absorbed in the small intestine [49]. Most FA, like other plant-derived hydrocinnamates, is ester-linked to plant cell wall polymers (cellulose, arabinox-ylan, and β-glucan) and cannot readily be absorbed in this complexed form [50, 51]. Esterase activity within the intestinal mucosa or the GMB must release the free FA before it can be absorbed and/or further metabolized [50–52]. In our study, urinary levels of FA were over 20-fold higher than those of catechin (Table 1) and did not vary across antibiotic treatment groups (Figure 1), suggesting that FA was highly bioavailable across treatment groups. In the rat, dietary FA makes a larger contribution to urinary SPAs than does free L-PHE or L-TYR [27, 53].

## ANTIBIOTIC EFFECTS ON THE GMB

Different classes of antibiotics produce different metabolomic patterns [8, 54], confirming some selectivity in the contributions that various constituents of the GMB make to the metabolome. An overview of our data provides additional detail. In the clindamycin and piperacillin/tazobac-tam groups, levels of 3,2-HPPA, 3,3-HPPA, and 3,4-HPPA were markedly lower than in controls (Figure 1). However, those same metabolites were elevated in the aztreonam-treated group, as were 2,3-DHHCA, 2,4-DHHCA, and 3,4-DHHCA. The findings starkly separate the 2 groups of antibiotics. In the rat, the excretion of urinary 3,3-HPPA is dramatically elevated when germ-free rats are inoculated with conventional GMB [24]. Broad spectrum antibiotic treatments lower levels of urinary 3,3-HPPA in mice [55] as well as levels of 3,3-HPPA [34, 55–57] and 3,4-HPPA [56] in rats. This makes the acute increases in 3,2-HPPA, 3,3-HPPA, and 3,4-HPPA (Figure 1) all the more striking. There are insufficient reports to comparably assess the levels of DHHCA. One group reported that a high bran diet, another source of FA, elevated urinary levels of a sulfat-ed-DHHCA isomer, which could not be fully resolved [58]. Our data would indicate that all the DHHCA isomers we measured are differentially sensitive to the GMB (Table 1). The accurate identification of the different DHHCA isomers is important for at least 2 reasons. First, they can be readily mistaken for other structural isomers [13]. Second, 3,4-DHHCA has been identified in rodent brain [59] where it can promote brain plasticity and attenuate some depression-like phenotypes [60].

Hippuric acid is an acyl glycine formed through the conjugation of benzoic acid with glycine [61]. Urinary hippuric acid levels are lower in germ-free mice than conventionally reared controls [1, 36], in mice acutely treated with vancomycin [62], and in rats acutely treated with a variety of antibiotics. This is attributed to GMB influences on benzoic acid synthesis [61]. Thus, the finding that clindamycin dramatically lowered urinary hippuric levels (Figure 1) is hardly surprising. However, in agreement with our earlier report [8], hippuric acid levels in the piperacillin/tazobac-tam or aztreonam-treated groups were not different from controls (Figure 1), providing additional separation between antibiotics.

This report is the first identification of 3,3-HPHPA in the mouse (Table 1). Of 4 groups that analyzed rat urine for the presence of 3,3-HPHPA, 2 confirmed its presence [24, 63] and 2 did not [32, 38]. One group reported that inoculation of germ-free rats with feces from specific-pathogen-free rats had no effect on urinary 3,3-HPHPA [24]. Similarly, we did not find any effect of antibiotic treatment on 3,3-HPHPA levels in the mouse (Figure 1). The oral antibiotic neomycin has been reported to dramatically lower excretion of 3,3-HPHPA in man [9, 64]. Whether species differences or other factors account for this is unclear. We evaluated female mice, in keeping with the larger body of work from our laboratory (CJD). Gender-specific effects of antibiotic on the GMB are certainly known [65]. Effects in male mice would need to be independently determined.

## CONCLUSIONS

Our data demonstrate that antibiotic treatments that target different groups of bacteria, produce significantly different urinary phenolic metabolomes (Figure 1). We previously showed that a course of piperacillin/tazobactam but not aztreonam disrupted colonization resistance to *C. difficile* [8]. Clindamycin is another antibiotic treatment that disrupts colonization resistance in mice [6]. The similarities in SPM changes in the clindamycin and piperacillin/tazobactam groups as opposed to the aztreonam-treated group and controls, raise the possibility that an elevation of urinary 3,2-HPPA, 3,3-HPPA, and 3,4-HPPA may reliably track antibiotic-induced colonization resistance. Such changes in the phenolic metabolome, combined with previously reported findings [8] may support the development of a clinical panel that could distinguish an intact flora from one vulnerable to overgrowth by pathogenic anaerobic bacteria. In support of this, longitudinal studies should be conducted that concurrently characterize the urinary phenolic metabo-lome, the fecal metabolome, the fecal microbiota, and functional measures of colonization resistance during the period of antibiotic treatment and recovery.

## Figures and Tables

**Supplementary Table 1.** Effects of antibiotics on colonic microbiota in the mouse.

Antibiotic	Clindamycin	Piperacillin / tazobactam	Aztreonam
Type	lincosamide	ureidopenicillin / β-lactam/β-lactamase inhibitor	monobactam
Suppression of indigenous microbiota	gram (-) and gram (+) anaerobes	gram (-) and gram (+) anaerobes, enterococci, and gram (-) facultative anaerobes (eg *E. coli*)	gram (-) facultative anaerobes (eg *E. coli*)
Promotion of pathogens	*C. difficile*, vancomycin-resistant enterococci, and facultative gram (-) bacilli (eg, *Klebsiella pneumoniae*)	*C. difficile*, vancomycin-resistant enterococci, and facultative gram (-) negative bacilli (eg, *Klebsiella pneumoniae*)	-
